# Decidual CXCR4^+^CD56^bright^NK cells as a novel NK subset in maternal–foetal immune tolerance to alleviate early pregnancy failure

**DOI:** 10.1002/ctm2.540

**Published:** 2021-10-14

**Authors:** Yu Tao, Yan‐Hong Li, Di Zhang, Ling Xu, Jia‐Jia Chen, Yi‐Fei Sang, Hai‐Lan Piao, Xue‐Ling Jing, Min Yu, Qiang Fu, Sheng‐Tao Zhou, Da‐Jin Li, Mei‐Rong Du

**Affiliations:** ^1^ NHC Key Lab of Reproduction Regulation (Shanghai Institute of Planned Parenthood Research), Hospital of Obstetrics and Gynecology Fudan University Shanghai Medical College Shanghai China; ^2^ Shanghai Key Laboratory of Female Reproductive Endocrine Related Diseases Shanghai China; ^3^ Department of Assisted Reproduction Shanghai Ninth People's Hospital Shanghai Jiao Tong University School of Medicine Shanghai People's Republic of China; ^4^ Department of Immunology Binzhou Medical College Yantai People's Republic of China; ^5^ Key Laboratory of Birth Defects and Related Diseases of Women and Children of MOE and State Key Laboratory of Biotherapy, Department of Obstetrics and Gynecology, West China Second University Hospital Sichuan University and Collaborative Innovation Center Chengdu People's Republic of China; ^6^ Department of Obstetrics and Gynecology, Guangzhou First People's Hospital, School of Medicine South China University of Technology Guangzhou China; ^7^ State Key Laboratory of Quality Research in Chinese Medicine and School of Pharmacy Macau University of Science and Technology Macau China

**Keywords:** CXCR4^+^CD56^bright^NK cells, maternal–foetal immunotolerance, NK cell‐based immunotherapy, recurrent miscarriage

## Abstract

Natural killer (NK) cells preferentially accumulate at maternal–foetal interface and are believed to play vital immune‐modulatory roles during early pregnancy and related immunological dysfunction may result in pregnant failure such as recurrent miscarriage (RM). However, the mechanisms underlying the establishment of maternal–foetal immunotolerance are complex but clarifying the roles of decidual NK (dNK) cells offers the potential to design immunotherapeutic strategies to assist RM patients. In this report, we analysed RNA sequencing on peripheral NK (pNK) and decidual NK cells during early pregnancy; we identified an immunomodulatory dNK subset CXCR4^+^CD56^bright^dNK and investigated its origin and phenotypic and functional characteristics. CXCR4^+^CD56^bright^dNK displayed a less activated and cytotoxic phenotype but an enhanced immunomodulatory potential relative to the CXCR4 negative subset. CXCR4^+^CD56^bright^dNK promote Th2 shift in an IL‐4‐dependent manner and can be recruited from peripheral blood and reprogramed by trophoblasts, as an active participant in the establishment of immune‐tolerance during early pregnancy. Diminished CXCR4^+^ dNK cells and their impaired ability to induce Th2 differentiation were found in RM patients and mouse models of spontaneous abortion. Moreover, adoptive transfer of CXCR4^+^ dNK cells to NK‐deficient (Nfil3^–/–)^ mice showed great therapeutic potential of CXCR4^+^ dNK via recovering the Th2/Th1 bias and reducing embryo resorption rates. The identification of this new dNK cell subset may lay the foundation for understanding NK cell mechanisms in early pregnancy and provide potential prognostic factors for the diagnosis and therapy of RM.

## Graphical abstract

Decidual CXCR4+ NK cell subset is recruited from peripheral blood and reprogramed by trophoblasts. CXCR4+ dNK cells featured with low activity and cytotoxicity but high capacity of inducing Th2 differentiation, are benefit to pregnancy immunotolerance. CXCR4+ dNK cell subset decreases in miscarriage and is less potent to mediate immune tolerance. Adoptive transfer of CXCR4+ dNK cells alleviates pregnancy loss.

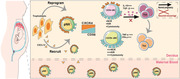

## 1 INTRODUCTION

Allogeneic tissue grafts are often rapidly rejected, requiring immunosuppressive medications after transplantation surgery.^1^ However, the semi‐allogeneic foetus in normal pregnancy or the total allogeneic foetus in surrogate pregnancy is generally accepted by the maternal immune system. The failure of maternal–foetal immune tolerance could lead to abnormal pregnancies including recurrent miscarriage (RM).^2^, ^3^ RM, defined as two or more consecutive pregnancy losses before 20 weeks, approximately affects 1–3% of women and has become one of the most frustrating problems in reproductive medicine.^4^ The pathogenesis of RM is complex and dysregulated maternal–foetal immunotolerance is believed to be involved.^5^, ^6^ Current clinical immunotherapies such as lymphocyte active immunotherapy (LIT) and intravenous immunoglobulin (IVIg) for RM are inconsistent and controversial.^7^, ^8^, ^9^ Therefore, new immunomodulatory therapies are urgently needed.

Multiple mechanisms were found to be potentially involved in immunological tolerance at maternal–foetal interface during early pregnancy.^10^ Recent research showed that trophoblasts produce thymic stromal lymphopoietin (TSLP), which induces dendritic cell‐mediated type‐2 T helper cell (Th2) bias and regulatory T‐cell (Treg) expansion in the decidua.^11^ Furthermore, immunoregulatory molecules, including interleukin (IL)‐10 and galectin‐9, play important roles in maternal–foetal immune tolerance.^12^, ^13^ Natural killer (NK) cells, a major contributor to innate immunity, have been shown to possess heterogeneity and plasticity involved in multiple progresses during pregnancy.^14^, ^15^, ^16^ Intriguingly, one of the most striking features of early pregnancy is the dramatic infiltration of NK cells.^17^ Our previous published data showed that CD56^bright^CD25^+^ NK cells distribute at the maternal–foetal interface preferentially and were conducive to immunotolerance during early pregnancy.^18^ Notably, in patients with RM, it has been shown that NK cells display enhanced cytotoxicity and dampened immunomodulatory capacity.^13^, ^19^, ^20^, ^21^, ^22^ Thus, a better understanding of NK cells will provide opportunities to design novel immunotherapeutic strategies for RM.

CXC chemokine receptor 4 (CXCR4), the receptor of chemokine CXCL12, is involved in homing and chemotaxis in the hematopoietic and immune systems. CXCR4 signalling regulates multiple processes including immune responses, vascular formation and morphogenesis,^23^, ^24^, ^25^ and accordingly, the CXCR4/CXCL12 axis constitutes a therapeutic target in multiple diseases.^26^, ^27^, ^28^ Our previous study has elucidated that CXCR4/CXCL12 can recruit peripheral NK (pNK) cells to the decidua and facilitates Th2 bias and maternal–foetal immune tolerance.^29^ And recently, single‐cell sequencing identified a subgroup of CD103^+^CD160^+^CD161^+^CD127^–^ NK cells (dNK3). This subgroup of NK cells was believed to play important roles in regulating extravillous trophoblast (EVT) invasion. They also found CXCR4 was highly expressed on dNK3 cells but no further research was done.^15^ Despite the abundant expression of CXCR4/CXCL12 at maternal–foetal interface,^30^ the modulatory effect of CXCR4 on decidual NK cells remains unclear. Similarly, whether aberrant CXCR4 expression on dNK cells is relevant to RM has not yet been reported. Answers to these questions are expected to promote CXCR4 as a candidate target for NK cell manipulations for clinical use during pregnancy.

In this study, we identified a subgroup of NK cells, CXCR4^+^CD56^bright^ dNK cells (abbreviated to CXCR4^+^ dNK), which originated from maternal peripheral blood by the recruitment of trophoblasts. This subset represents a relatively inactivated phenotype but potent production of anti‐inflammatory cytokines. CXCR4^+^ dNK cells are the main source of interleukin‐4 (IL‐4) and possess the ability to keep immune tolerance by inducing Th2 bias at maternal–foetal interface. The unique immunomodulatory actions of CXCR4^+^ dNK cells were further confirmed in both animal models and RM patients. Most importantly, the therapeutic potential of CXCR4^+^ dNK cells was established by the adoptive transfer of this NK subset into pregnant NK‐deficient (Nfil3^–/–^) mice, proposing a promising NK cell‐based immune‐therapeutic strategy for clinical use.

## 2 MATERIALS AND METHODS

### 2.1 Human sample collection

First‐trimester villi and decidua were collected from healthy pregnant women together with peripheral blood for peripheral blood mononuclear cell (PBMC) isolation. Other decidua was collected from women with unexplained recurrent miscarriages (RM) at first trimester of pregnancy (Supplemental Table S1). For the RM group, all subjects have regular menstrual cycles and a history of two or more miscarriages with the same partner before 20 weeks. In the control normal pregnancy (NP) (Supplemental Table S2) group, foetal heartbeat was confirmed at 6–10 gestational weeks by ultrasound before the termination of pregnancy for non‐medical reasons. All NP women previously had at least one live birth and exhibited no spontaneous miscarriage. Excluding factors were: (a) genetic abnormalities and reproductive organ anomaly; (b) reproductive tract infections; (c) endocrine dysfunction and systemic diseases; (d) unhealthy lifestyle (e.g. drugs). Endometrial samples were collected from 30 healthy fertile women at the proliferative or secretory phase of their normal menstrual cycle (Supplemental Table S3). All tissues were immediately carried to the experiment bench for analysis within 30 min after operation and kept in ice‐cold Dulbecco's Modified Eagle Medium (DMEM) plus DMEM/F12 or high D‐glucose (Gibco, Grand Island, NY, USA). Magnesium and calcium‐free Hank's Balanced Salt Solution (HBSS) was used for tissue washes and cell isolation. All study procedures involving human specimens were approved by the Human Research Ethics Committee, Fudan University Obstetrics and Gynecology Hospital, Shanghai, China.

### 2.2 Isolation and primary culture of trophoblasts

Human villous tissues were separated from the decidua and cut into small pieces carefully to isolate trophoblasts according to previously described methods.^31^ Briefly, placental tissues were digested by 0.25% trypsin and 0.02% DNase type I at 37°C with subtle vibration for 5 min. Then the upper layer of the suspension was discarded, and the remaining part was collected. Totally, four cycles of digestion were completed. Trypsin digestion was stopped by the addition of 10% foetal bovine serum (FBS) and the digestions pooled and centrifuged at 250 × *g* for 10 min, before re‐suspension in 4 ml DMEM‐high glucose. The resulting solution was loaded onto a discontinuous 5%–70% Percoll gradient (vol/vol) in 5% steps of 2 ml each and centrifuged at 600 × *g* for 20 min. Cells at densities between 1.048 and 1.062 g/ml were collected and incubated in 5% CO_2_ at 37°C for 15 min to exclude contaminating cells. Non‐adherent cells were collected and cultured in DMEM‐high glucose with 15% FBS medium at 37°C in 95% air and 5% CO_2_. Trophoblast conditioned medium (TCM) was collected after 72 h culture and passed through a 0.22 μm filter and kept at −20°C for storage and use.

### 2.3 Isolation and culture of immune cells from deciduas

Decidual immune cells (DICs) were digested by trypsin‐DNase I and subjected to Percoll gradient centrifugation as previously described.^18^ Cells at density between 1.062 and 1.077 g/ml were harvested and cultivated in Roswell Park Memorial Institute (RPMI) 1640 plus 10% FBS medium in 5% CO_2_ at 37°C overnight before collection of the non‐adherent DICs. NK cell enrichment from the DICs was then performed using the Human NK cell magnetic activated cell sorting (MACS) kit (Miltenyi Biotec, Auburn, CA, USA). To separate CXCR4^+^ and CXCR4^–^ dNK cells, NK cells were first labelled with CXCR4‐Biotin antibody (Order no. 130098348, Miltenyi Biotec, Auburn, CA, USA) before subsequent incubation with Anti‐Biotin MicroBeads (Order no. 130‐090‐485, Miltenyi Biotec, Auburn, CA, USA) and positive selection of CXCR4^+^ NK cells to yield CXCR4^+^ and CXCR4^–^ NK cell subsets using magnetic separation. Purified CXCR4^+^ or CXCR4^–^ dNK cells seeded in 24‐well plates at a density of 2 × 10^5^ cells/ml per well in the presence of IL‐15 (10 ng/ml, PeproTech, USA) were treated with recombinant human CXCL12 (rhCXCL12, 100 ng/ml, R&D Systems) alone or in combination with CXCR4 antagonist AMD3100 (50 μmol/L, Tocris Bioscience) for 48 h. Cells were stimulated with Brefeldin‐A (10 mg/ml), phorbol myristate acetate (PMA) (50 ng/ml) and ionomycin (1 μg/ml) for intracellular cytokine analysis by flow cytometry (FCM) 4 h before harvested.

### 2.4 Purification of immune cells from human peripheral blood

Density centrifugation was performed to isolate PBMCs from whole blood using Ficoll‐Hypaque (Amersham Biosciences, USA) as previously described.^18^ Briefly, peripheral blood (30 ml) supplemented with anticoagulant was first diluted with same volume of phosphate buffered saline (PBS). Second, the same volume of Ficoll density gradient medium was used. The mixture was then centrifuged for 20 min at 600 × *g* and PBMCs collected and washed two or three times in PBS with 10 min at 250 × *g* centrifugation steps. CXCR4^+^ and CXCR4^–^ pNK cells were separated from PBMC as described above (Section 2.3). A MACS human naïve CD4^+^ T‐cell isolation kit (Miltenyi Biotec) was used to isolate naïve CD4^+^ T cells from PBMCs.

### 2.5 Allogeneic co‐culture of trophoblasts and pNK cells

Freshly isolated trophoblasts were seeded in 24‐well plates (2 × 10^5^ cells/ml per well) overnight and the adherent trophoblasts thoroughly washed with cold PBS. MACS‐purified pNK cells (2 × 10^5^ cells) were added to the trophoblast culture system or cultured alone for 72 h before recovering the pNK cells in the cell supernatants. Brefeldin‐A (10 mg/ml), PMA (50 ng/ml) and ionomycin (1 μg/ml) were used for intracellular cytokine analysis. NK cells were harvested and analysed for the expression of membrane molecules and intracellular cytokines by FCM after centrifugation at 250 × *g* for 10 min. CXCR4^+^ and CXCR4^–^ pNK cells were purified by MACS and labelled with 5‐(and 6)‐carboxyfluorescein diacetate, succinimidyl ester (CFSE, green fluorescent dye) or PKH26 (red fluorescent dye). CFSE‐CXCR4^+^ pNK cells (2 × 10^5^ cells), PKH26‐CXCR4^–^ pNK cells (2 × 10^5^ cells) and a mixture of these two labelled pNK subsets (cell number of the mixture was 2 × 10^5^ and CXCR4^+^: CXCR4^–^ pNK = 9:1) were co‐cultured with trophoblasts (2 × 10^5^ cells), respectively. After 72 h, all of the co‐cultured cells were harvested and analysed using FCM.

### 2.6 Induction of human Th2 cells

Co‐cultures of naïve CD4^+^ T cells with CXCR4^+^ or CXCR4^–^ dNK cells from first‐trimester pregnant women were performed. Naïve CD4^+^ T cells were first isolated from PBMCs via a MACS CD4^+^ T‐cell isolation kit (Miltenyi Biotec) before cultured in RPMI 1640 medium 96‐well round‐bottom plates for 5 days (1 × 10^5^ cells per well). T cells were cultured in the absence or presence of CXCR4^+^ or CXCR4^–^ dNK cells at 1:1 ratio with or without anti‐IL‐4 antibody (10 μg/ml, eBioscience). Cells were activated by plate‐bound CD3 (5 μg/ml; BD Biosciences, USA) and CD28 (1 μg/ml; BD Biosciences) antibodies. To sustain NK cells in culture, IL‐15 (10 ng/ml; PeproTech, USA) was used in the co‐culture system. After 5‐day culture, CD4^+^ T cells were pre‐treated for 4 h with PMA (50 ng/ml), ionomycin (1 μg/ml) and Brefeldin A (10 μg/mL) before collection and analysis by FCM.

### 2.7 Chemotaxis assay

Chemotaxis was assessed as previously described.^31^ Briefly, the upper well of Transwell plates (24‐well, 5.0 μm pore size; Corning, USA) were seeded with purified pNK suspensions (10^6^ cells/200 μl) while the bottom chamber contained TCM (800 μl) with or without anti‐CXCR4 (1 μg/ml; R&D Systems, USA), or control medium with or without rhCXCL12 (R&D Systems, USA). Cells migrating into the lower chamber after 3 h at 37°C were isolated and labelled with fluorescence‐conjugated antibodies. The absolute number of chemotactic cells was determined by FCM as described.^31^

### 2.8 RNA‐seq analysis

Total RNA was isolated from trophoblasts or purified NK cells using TRIzol according to the manufacturer's instructions. After purification, RNA samples were reverse transcribed into cDNA and fragmented before 3′ adenylation and adaptor ligation to construct libraries using the TruSeq® RNA LT Sample Prep Kit v2 (Illumina). Sequencing was conducted by Genergy Biotechnology Co. Ltd. (Shanghai, China) on the Illumina HiSeq X Ten instrument. Original data were generated in FASTQ format (read length 2 × 150 bp). In each sample, number of transcripts was calculated as fragments per kilobase per million (FPKM); Cuffnorm and DESeq software was used to analyse FPKM and differential gene transcripts (DETs). *p* < .05 and absolute fold change ≥2 were set for thresholds for determining DETs.

### 2.9 Flow cytometry

Cell surface molecular expression and intracellular cytokine production were analysed using FCM. A total of 10 000 events (minimum) were acquired via a flow cytometer (Beckman‐Coulter CyAN ADP) with analysis using FlowJo software (Tree Star, Ashland, OR, USA). Cell surface staining was performed with flurochrome conjugated monoclonal antibodies for 30 min incubation. After fixation and permeabilisation, cells were stained for intracellular cytokines and nuclear transcription factors. Antibodies were purchased from Biolegend, USA or eBioscience, USA (Supplemental Table S4). Isotype‐matched IgG reagents were used as negative controls. Fluorescence‐activated cell sorting was performed according to the manufacturer's instructions.

### 2.10 Abortion‐prone murine models

Male BALB/c mice (8–10 weeks) were obtained from Laboratory Animal Science Department, Fudan University (Shanghai, China). Female CBA/J (8–10 weeks) and male DBA/2mice (8–10 weeks) were obtained from Beijing HFK Bioscience Co., Ltd (Beijing, China). Animals were all raised in specific pathogen‐free environment. To establish normal and the abortion‐prone pregnancy models, female CBA/J mice were mated with either male BALB/c or DBA/2 mice, respectively.^32^, ^33^ E0.5 was defined as the day of detection of a vaginal plug. For the NK depletion assay, female CBA/J were mated with male BALB/c, and pregnant CBA/J were intraperitoneally injected with 30 μl anti‐asialo GM (ASGM)‐1 (NK depletion agent, Wako Chemicals, Japan) in 200 μl PBS or 200 μl PBS alone as a control on E0.5, E3.5, E6.5 and E8.5. Mice were euthanised on E10.5 to examine embryo resorption rate and cytokine expression. For rescue experiments, 5 μg recombinant murine IL‐4 in 100 μl PBS or 100 μl PBS alone was injected into NK cell‐depleted CBA/J pregnant mice via the tail vein on E4.5, E6.5 and E8.5. Mice were euthanised on E10.5 to examine embryo resorption rate and cytokine expression.

### 2.11 Adoptive transfer of CXCR4^+^ NK cells to NK‐deficient mouse

The origin and phenotype of Nfil3^–/–^ mice have been previously described; and this transcription factor defect leading to a failure of NK cell development and function.^34^ Nfil3^–/–^ mice were provided by Professor H. Wei. Female Nfil3^–/–^ mice or wide‐type (WT, C57BL/6) were mated with male C57BL/6 mice. Decidual CXCR4^+^ NK cells and CXCR4^–^ NK cells were isolated from pregnant WT mice (E7.5) by fluorescence‐activated cell sorting (FACS) and were labelled with the fluorescent dyes Did and Dir, respectively. Did‐CXCR4^+^ dNK cells or Dir‐CXCR4^–^ dNK cells (3 × 10^5^) were resuspended in PBS (200 μl) and injected into pregnant Nfil3^–/–^ mice via the tail vein at E7.5. PBS was injected into pregnant Nfil3^–/–^ mice at E7.5 as controls. In vivo optical imaging was performed after 3 days to detect the labelled NK cells. At E10.5, the experimental mice were sacrificed and embryo resorption rate was calculated.

All study procedures involving animals were approved by the Human Research Ethics Committee of Obstetrics and Gynecology Hospital of Fudan University.

### 2.12 Quantification of embryo resorption

Pregnant mice were euthanised and analysed for the resorption of embryos on E10.5. The clinical manifestation of resorbing embryos at this stage were smaller and darker embryos due to haemorrhage, ischemia and necrosis compared to normal viable, larger, pink, healthy embryos. The definition of embryo resorption rate was the number of embryos resorbed divided by the total number of healthy embryos × 100%.

### 2.13 Statistical analysis

Statistical software Prism 7.0 was employed for data analysis. The significance of differences between two groups was determined by *t* test. Multiple groups were analysed via one‐way or two‐way ANOVA with Bonferroni's post hoc test. Statistically significance was set as *p* value < .05 for all tests.

## 3 RESULTS

### 3.1 Decidual‐specific CXCR4^+^CD56^bright^ NK cells are preferentially enriched in early pregnancy

**FIGURE 1:**
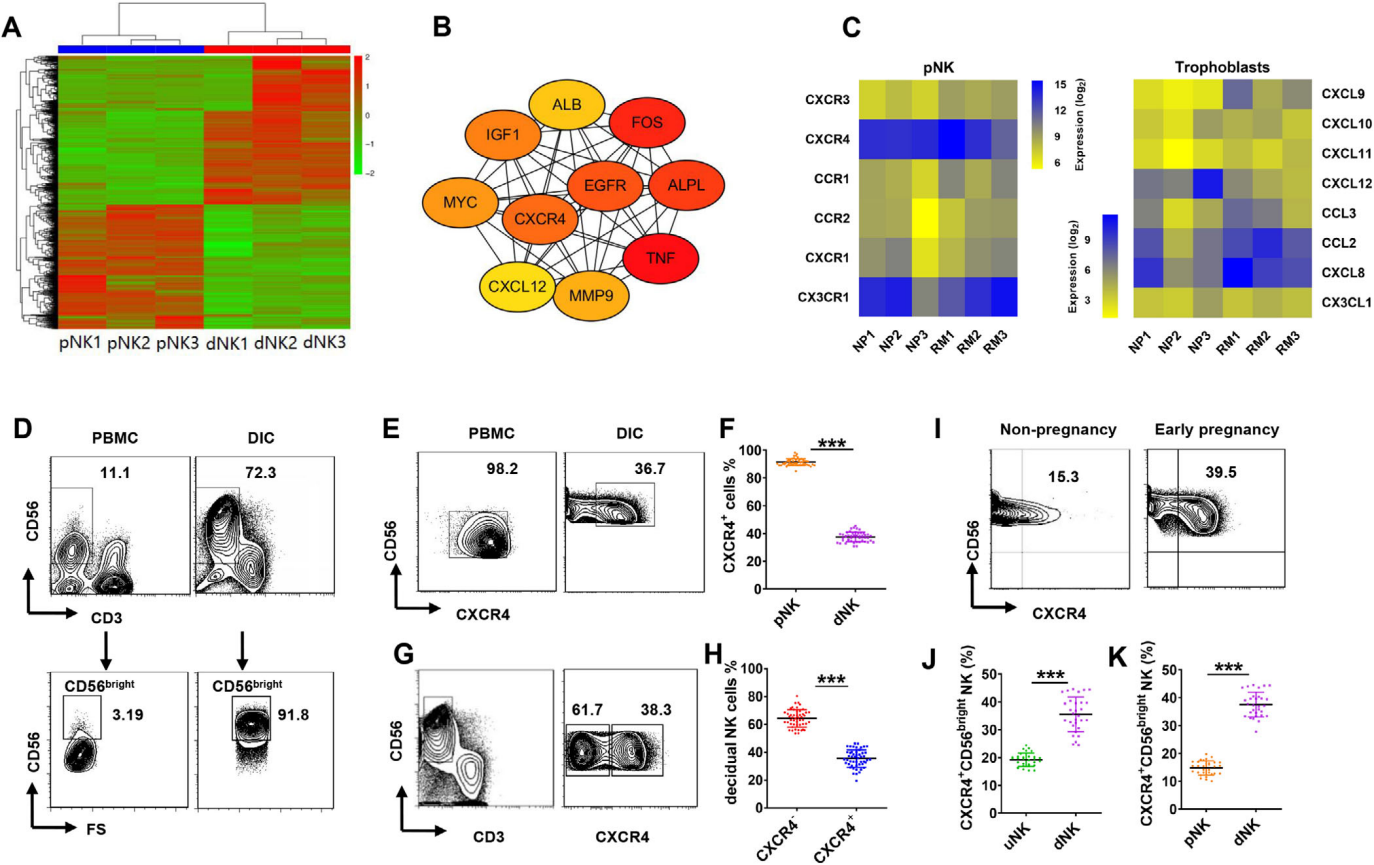
CXCR4^+^CD56^bright^ NK accumulate in the decidua preferentially during early pregnancy. pNK cells and dNK cells purified from normal first trimester pregnant women and RNA‐seq analysis was performed. (A) Heatmap showing differentially expressed genes (DEGs) between dNK (*n* = 3) and pNK cells (*n* = 3). (B) Analysis of the 10 most important hub genes using the cytoscape software plugin cytoHubba. (C) pNK cells and trophoblasts derived from normal pregnant women (*n* = 3) and RM patients (*n* = 3) and RNA‐seq analysis was performed. Heatmaps showing log‐transformed, normalised gene expression of selected chemokine receptors on pNK cells (left) and corresponding chemokines in trophoblasts (right) between NP and RM. (D) Percentage of CD56^bright^ NK in gated CD56^+^CD3^–^ NK isolated from PBMCs and DICs during first trimester. (E and F) Percentage of CXCR4^+^ NK in gated CD56^+^CD3^–^ NK isolated from PBMCs (pNK) and DICs (dNK) during first trimester. *n* = 50 for pNK and dNK cells, respectively. (G and H) dNK cells divided into two subpopulations according to CXCR4 expression. *n* = 50. (I and J) More CXCR4^+^CD56^bright^ NK cells were distributed in the decidua of first‐trimester women than uterine endometrial NK (uNK) cells in non‐pregnant women. *n* = 30 for uNK and dNK cells, respectively. (K) Percentage of CXCR4^+^CD56^bright^ NK cells in pNK and dNK. *n* = 30 for pNK and dNK cells, respectively. In all figures, data are presented as mean ± standard error of the mean (SEM). (***p < .001; Student's test)

To identify key regulators of dNK cells and their underlying mechanisms in maternal immune‐tolerance, we performed RNA‐sequencing analysis to compare first trimester peripheral and decidual NK cells from the same pregnant women along with their trophoblasts. Consistent with previous research,^35^ the gene expression profile of dNK cells was clearly distinct from pNK cells (Figure 1A). The protein–protein interaction (PPI) network of differentially expressed gene (DEGs) (Figure S1) and hub genes analysis using Cytoscape suggested that CXCR4 and CXCL12 were potential regulatory factors of dNK cells (Figure 1B). Moreover, pNK cells were shown to express high levels of CXCR4 and CX3C motif chemokine receptor 1 (CXC3CR1) in normal pregnancy (Figure 1C). Correspondingly, CXCL12, the ligand of CXCR4, was the most enriched chemokine gene in normal trophoblasts while only very low levels of CX3C motif chemokine ligand 1 (CX3CL1), the ligand of CXC3CR1 were expressed. In addition, all the chemokine receptors on pNK cells, except CXCR4 and CXC3CR1, were higher in RM. Moreover, the expression of all corresponding chemokines was higher in trophoblasts from RM patients, while CXCL12 was the only lowered chemokine gene (Figure 1C).

We then focused on the regulation of CXCR4 and CXCL12 on dNK cells. Comparisons between peripheral and decidual NK cells showed more than 90% of dNK cells were CD56^bright^ NK; however less than 5% of pNK (∼1% of total lymphocytes) were CD56^bright^ NK cells (Figure 1D). Further analysis showed that 90% of pNK cells expressed CXCR4, whereas only around 40% of dNK cells were CXCR4 positive. Although most pNK cells expressed CXCR4, they were mainly CXCR4^+^CD56^dim^ NK cells (Figure 1E and F). FCM analysis showed that dNK cells could be subgrouped into two discrete populations based on CXCR4 expression (∼40% CXCR4^+^ and ∼60% CXCR4^–^; Figure 1G and H). In addition, comparisons of CXCR4^+^CD56^bright^NK cells in early pregnancy and non‐pregnancy states showed that CXCR4^+^CD56^bright^NK cells were mainly distributed in the decidua of first trimester pregnancy compared to endometrial NK cells of normal non‐pregnancy and peripheral NK cell of first trimester pregnancy (Figure 1I and K). Together, these data demonstrate a subset of decidual‐specific CXCR4^+^CD56^bright^NK cells preferentially accumulate in the first trimester of pregnancy.

### 3.2 CXCR4^+^ dNK cells display less activation characteristics but more immunomodulatory potency than CXCR4^–^dNK cells

**FIGURE 2:**
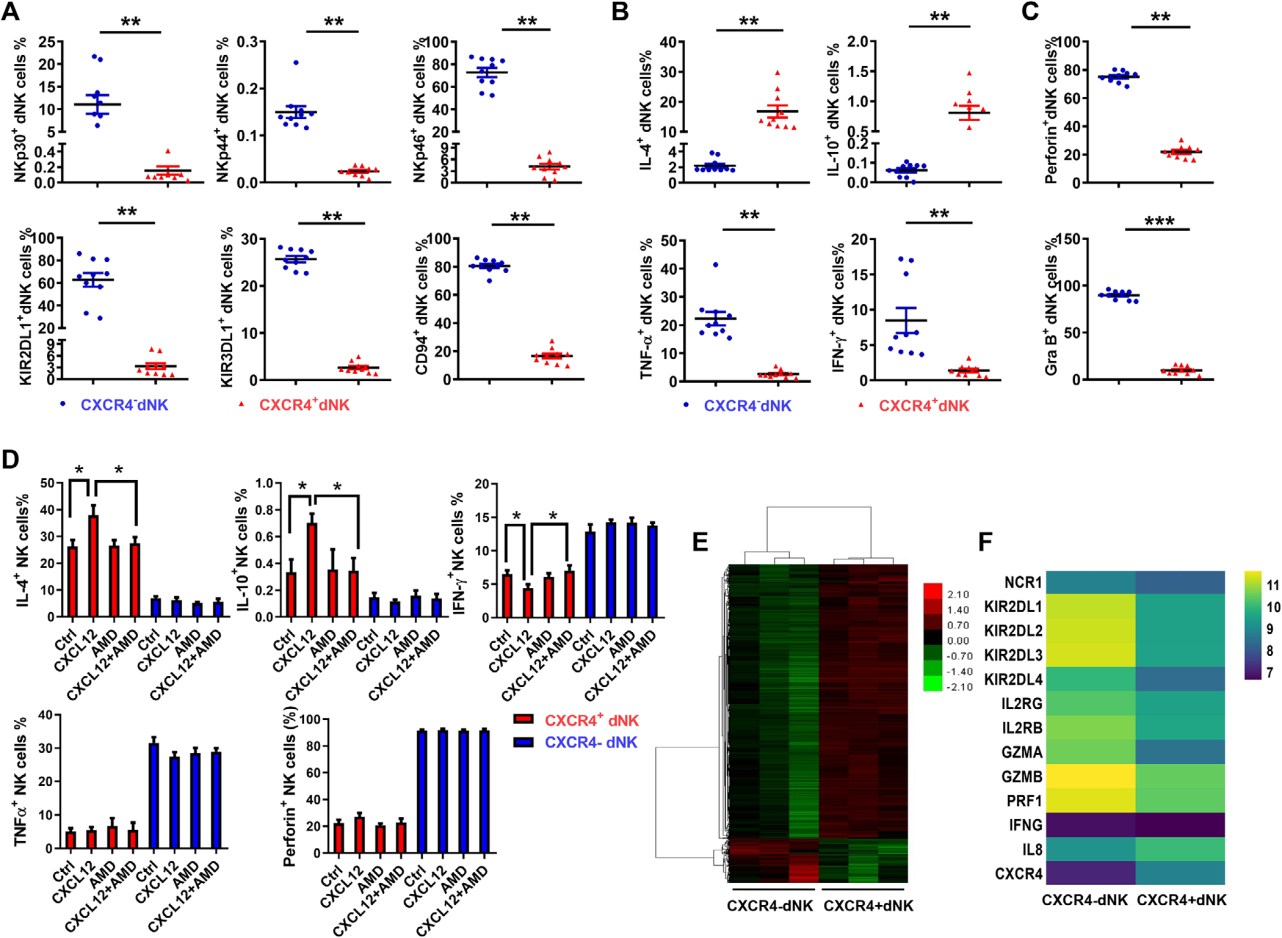
CXCR4^+^ dNK cells represents a subset with less activated and cytotoxic characteristics but immunomodulatory potential. (A) Expression of activating (NKp30, NKp44 and NKp46) and inhibitory receptors (KIR2DL1, KIR3DL1 and CD94) on CXCR4^+^ and CXCR4^–^ dNK cells. *n* = 10. (B) Production of cytokines including IL‐4, IL‐10, TNF‐α, IFN‐γ by CXCR4^+^ and CXCR4^–^ dNK cells. *n* = 10. (C) Cytotoxic markers perforin and granzyme B expressed in CXCR4^+^ and CXCR4^–^ dNK cells. *n* = 10. (D) Expression of cytokines and perforin in sorted CXCR4^+^ and CXCR4^–^ dNK cells treated with CXCL12 alone or in combination with the inhibitor, AMD3100. (E) Heatmap showing differentially expressed gene profiles of sorted CXCR4^+^ and CXCR4^–^ dNK cells (*n* = 3, 3). (F) Heatmap showing log‐transformed, normalised expression level of selected genes in CXCR4^+^ and CXCR4^–^ dNK cells (*n* = 3, 3). The data presented are mean ± SEM. (*p < .05, **p < .01, ***p < .001; Student's test)

We next compared the phenotype and function of CXCR4^+^ dNK cells and CXCR4^–^ dNK cells. Notably, both the activating (NKp30, NKp44 and NKp46) and inhibitory (KIR2DL1, KIR3DL1 and CD94) receptors were more lowly expressed on CXCR4^+^ dNK cells relative to CXCR4^–^ dNK cells (Figure 2A). Profiling of the cytokine production by the two NK subsets revealed anti‐pro‐inflammatory cytokines such as IL‐4, IL‐10, IL‐8, IL‐22, TGF‐β were significantly higher in CXCR4^+^ dNK cells while pro‐inflammatory cytokines TNF‐α and IFN‐γ were relatively higher in CXCR4^–^ dNK cells (Figure 2B and S2). However, there were no differences in their production of IL‐12, IL‐17A and IL‐13 (Figure S2). Examination of cytotoxic marker levels also showed that CXCR4^+^ dNK cells expressed lower levels of perforin and granzyme B than CXCR4^–^ dNK cells (Figure 2C). Moreover, the preferential expression of anti‐inflammatory cytokines in CXCR4^+^ dNK cells could be promoted by treatment with rhCXCL12 (Figure 2D). Here rhCXCL12 treatment contributed to higher production of IL‐4 and IL‐10 but lower production of INF‐γ in CXCR4^+^ dNK cells, which was abrogated by CXCR4 antagonist, AMD3100. This effect mediated by rhCXCL12 was not achieved on CXCR4^–^ dNK cells (Figure 2D). Thus, the distinguished phenotype and functions of the two decidual NK cell subtypes are defined by CXCR4 expression during early pregnancy.

Finally, RNA‐sequencing analysis was used to confirm the differences between CXCR4^+^ and CXCR4^–^ dNK cells (Figure 2E). Indeed, the gene expression profile of the CXCR4^+^ subset greatly differed from that of CXCR4^–^ subset. Specially, genes encoding activating and inhibitory receptors, pro‐inflammatory cytokines and cytotoxic factors were less enriched in the CXCR4^+^ subset compared to the CXCR4^–^ subset (Figure 2F). Taken together, CXCR4^+^ dNK cells represent a shift away from the activated, cytotoxic phenotype towards cells with higher immunomodulatory potential.

### 3.3 Human trophoblasts recruit and reprogram peripheral NK cells into CXCR4^+^CD56^bright^ dNK cells during early pregnancy

**FIGURE 3:**
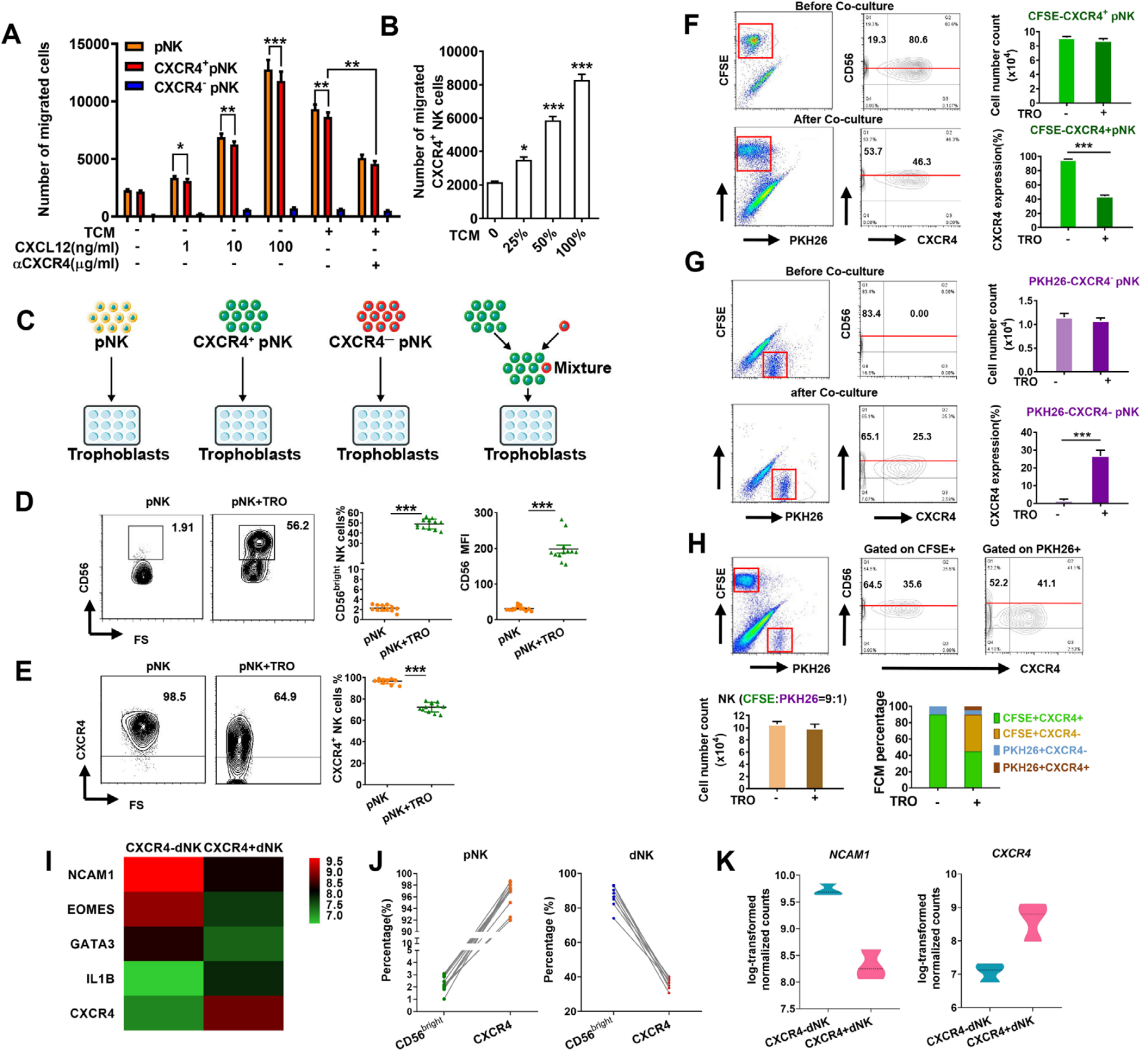
CXCR4^+^ dNK cells originate from pNK cells recruited and reprogrammed by trophoblasts. (A and B) Trophoblasts exert chemotactic effects on CXCR4^+^ pNK cells via CXCL12. *n* = 6 (C) Schematic for the co‐culture of trophoblasts with equal number of total pNK cells, CFSE‐labelled CXCR4^+^ pNK cells, PKH26‐labelled CXCR4^–^ pNK cells or the mixture of these two labelled NK subsets. FCM was performed after 72 h co‐culture. (D and E) Percentage and mean fluorescent intensity (MFI) of CD56 (D) and the percentage of CXCR4 (E) on pNK cells cultured alone or with trophoblasts. (F) Number and frequency of CXCR4^+^ pNK cells in the CFSE‐labelled CXCR4^+^ pNK cells and trophoblast co‐culture system. (G) Number and frequency of CXCR4^+^ pNK cells in the PKH26‐labelled CXCR4^–^ pNK cells and trophoblast co‐culture system. (H) Number and frequency of CXCR4^+^ pNK cells and CXCR4^–^ pNK cells in the mixture of labelled NK cells co‐cultured with trophoblasts. (I) Heatmap showing normalised expression of selected genes associated with NK development in CXCR4^+^ and CXCR4^–^ dNK cells (*n* = 3, 3). (J) The expression of CD56^bright^ and CXCR4 on pNK cells and dNK. *n* = 10. (K) Violin plots showed log‐transformed, normalised level of *NCAM1* and *CXCR4* expressed in CXCR4^+^ and CXCR4^–^ dNK cells. *n* = 3. Data are presented as mean ± SEM. (*p < .05, **p < .01, ***p < .001; Student's test)

Transwell migration assay was used to explore the likelihood of CXCR4^+^CD56^bright^ dNK cells originating from pNK cells. We found that rhCXCL12 significantly increased the number of pNK cells and that CXCR4^+^ pNK cells migrated to the lower Transwell chambers in a concentration‐dependent manner. Notably, trophoblast conditioned medium (TCM) also potently stimulated chemotaxis of total pNK cells and CXCR4^+^ pNK cells. Moreover, pre‐addition of CXCR4 antibodies in the upper well efficiently blocked the chemotactic properties of TCM (Figure 3A). Furthermore, TCM was capable of attracting large numbers of pNK cells, especially CXCR4^+^ pNK cells and its chemotactic activity was proportional to the dilution of TCM used (Figure 3B).

In order to further explore the origin of CXCR4^+^ dNK cells, pNK cells were co‐cultured with trophoblasts. Total pNK cells, CFSE‐labelled CXCR4^+^ pNK cells, PKH26‐labelled CXCR4^–^ pNK cells or the mix of labelled pNK cells (CXCR4^+^: CXCR4^–^ pNK = 9:1, mimicking the proportion of CXCR4^+^ and CXCR4^–^ pNK cells in total pNK cells), respectively, were added to cultured trophoblasts (Figure 3C and S3). Analysis by FCM showed that pNK cells co‐cultured with trophoblasts displayed significant upregulation of CD56 expression (Figure 3D) while CXCR4 expression was downregulated (Figure 3E). Moreover, after 72 h cultured with trophoblasts, a proportion of the CFSE‐labelled CXCR4^+^ pNK cells had lost CXCR4 expression (Figure 3F). Unexpectedly, we also found a proportion of the PKH26‐labelled CXCR4^–^ pNK cells differentiated into PKH26^+^CXCR4^+^ pNK cells (Figure 3G). The reciprocal conversion between CXCR4^+^NK cells and CXCR4^–^NK cells was also evident in the trophoblast co‐culture experiments using dual labelled mixtures of CXCR4^+^ and CXCR4^–^ pNK cells, ultimately leading to the diminished proportion of CXCR4^+^ pNK cells (Figure 3H). However, we observed no change in the absolute number of labelled CXCR4^+^, CXCR4^–^ or mixed pNK cells (Figure 3F and H). It was notable that increased CD56 expression accompanied the decreased expression of CXCR4 on CFSE‐labelled pNK cells (Figure 3F and H), which was also consistent with the changes seen in Figure 3D. These results suggested a possible association between the expression of CXCR4 and CD56.

We further analysed the expression of marker genes and transcriptional factors regulating NK cells maturation in the subsets defined by CXCR4 expression. *Neural cell adhesion molecule 1* (*NCAM1), Eomesodermin (EOMES)* and GATA binding protein 3 (*GATA3)*, believed to promote NK cells maturation, were more highly expressed in CXCR4^–^ dNK cells while *IL1B*, a negative regulator of NK cell maturation, exhibited increased expressed in CXCR4^+^ dNK cells (Figure 3I). The relationship of CD56 and CXCR4 expressed on pNK and dNK cells (Figure 3J) and the relationship of *NCAM1* (coding CD56) and *CXCR4* gene expression in CXCR4^+^ and CXCR4^–^ dNK cells (Figure 3K) clearly showed a negative correlation. Together, these results suggest that CXCR4^+^ dNK cells may originate from both of CXCR4^+^ and CXCR4^–^ pNK cells and be influenced by trophoblasts at the maternal–foetal interface.

**FIGURE 4:**
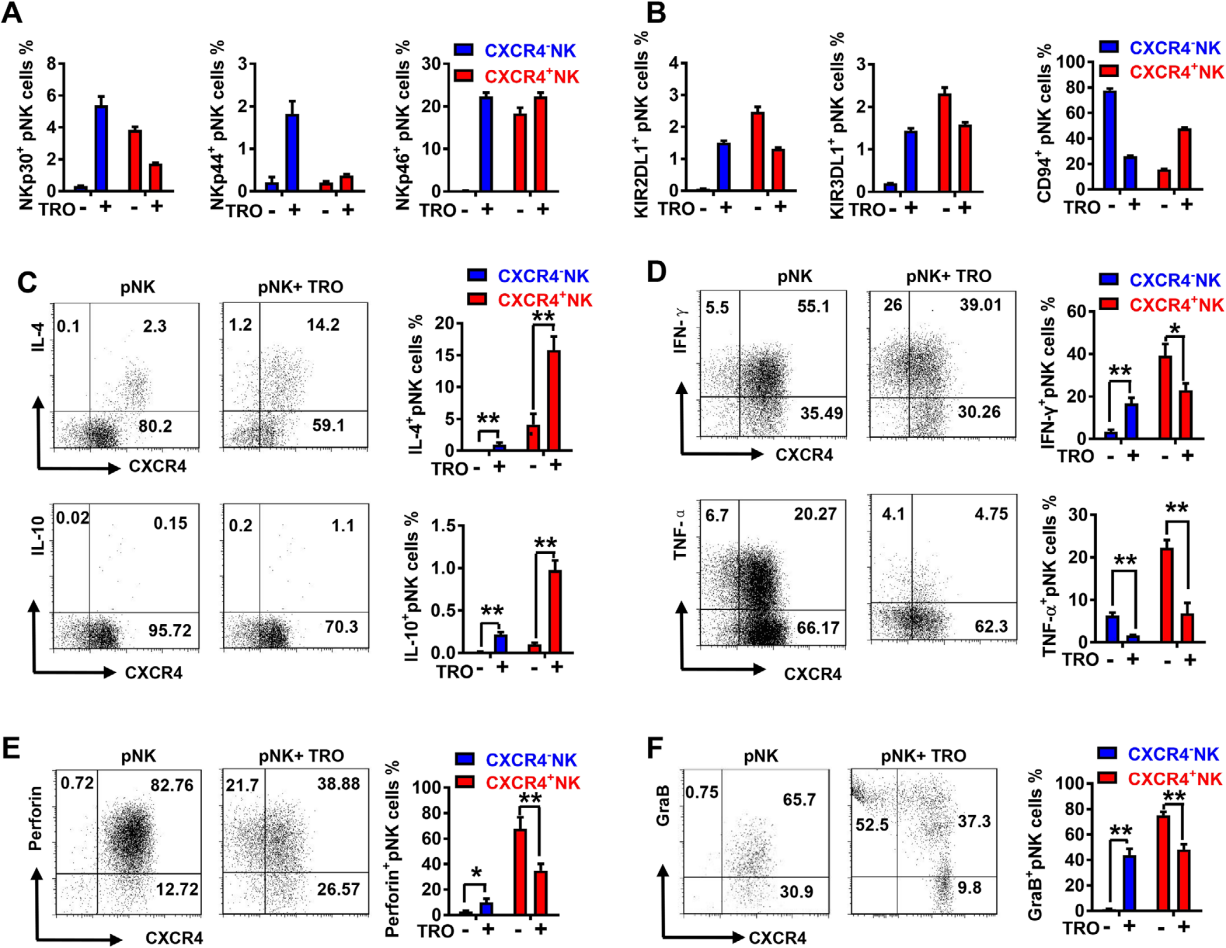
Human trophoblasts induce CXCR4^+^ pNK cells recruited from peripheral blood to adopt a dNK‐like phenotype. Purified pNK cells were cultured alone or with trophoblasts. FCM was performed to detect the expression of (A and B) excitatory (NKp30, NKp44 and NKp46) and inhibitory receptors (KIR2DL1, KIR3DL1, CD94), (C and D) anti‐inflammatory cytokines (IL‐4 and IL‐10) and pro‐inflammatory cytokines (IFN‐γ and TNF‐α), (E and F) Perforin and GraB in CXCR4^+^ pNK and CXCR4^–^ pNK cells. *n* = 10. The data presented are mean ± SEM. (*p < .05, **p < .01, ***p < .001; Student's test)

Besides the expression of CD56 and CXCR4, we explored other potential phenotypic and functional changes in pNK cells elicited by trophoblasts. As showed in Figure 4A and B, co‐culture with trophoblasts increased higher levels of the activating receptors, NKp30, NKp44 and NKp46 on CXCR4^–^ pNK, together with higher levels of inhibitory receptors KIR2DL1 and KIR3DL1 and lower levels of CD94 (Figure 4A and B). Conversely, CXCR4^+^ pNK cells exhibited lowered levels of NKp30, KIR2DL1 and KIR3DL1 and higher levels of CD94 in the trophoblasts co‐culture system (Figure 4A and B). Moreover, the expression of anti‐inflammatory cytokines IL‐4 and IL‐10 by CXCR4^+^ pNK cells were substantially increased by trophoblasts. Co‐culture with trophoblasts also increased the expression of IL‐4 and IL‐10 in CXCR4^–^ pNK cells, although to a lesser degree (Figure 4C and D). Furthermore, the production of pro‐inflammatory cytokine TNF‐α was reduced by trophoblasts in both CXCR4^+^ pNK cells and CXCR4^–^ pNK cells. Notably, trophoblasts reduced the production of IFN‐γ in CXCR4^+^ pNK cells but augmented IFN‐γ expression in CXCR4^–^ pNK cells (Figure 4C and D). We also analysed the effect of trophoblasts on the NK cell cytotoxicity. Co‐culture with trophoblasts upregulated perforin and granzyme B expression in CXCR4^–^ pNK cells but downregulated their expression in CXCR4^+^ pNK cells (Figure 4E and F). Together, these results suggest that trophoblasts reprogram peripheral NK cells towards CXCR4^+^ dNK cell‐like phenotype and function.

### 3.4 CXCR4^+^ dNK cells are conducive to maternal immune tolerance by promoting IL‐4‐mediated Th2 differentiation

**FIGURE 5:**
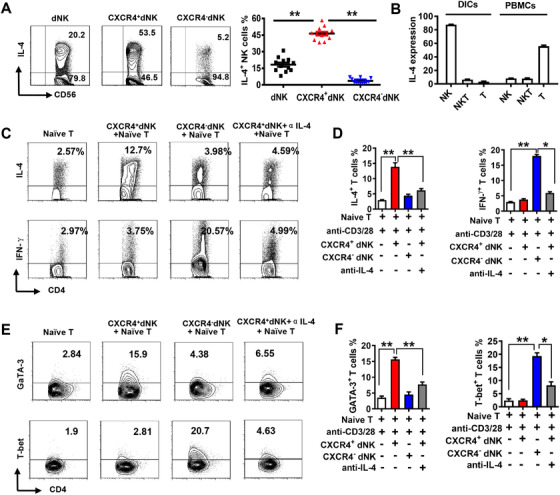
CXCR4^+^ dNK cells facilitates Th2 differentiation via IL‐4. (A) FCM analysis of IL‐4 expression in total dNK cells, CXCR4^+^ and CXCR4^–^ dNK subsets (*n* = 10). (B) Percentage of IL‐4‐expressing cells among PBMCs and DICs (*n* = 10). CXCR4^+^ dNK cells or pre‐treatment with anti‐IL‐4 and CXCR4^–^ dNK cells were added to the CD4^+^CD45RA^+^ naïve T cells induced differentiation system. FCM was performed to determine the differential outcomes by analyse the typical cytokines and transcriptional factors. Representative FCM plots (C) and statistical analysis (D) of Th2 typical cytokine IL‐4 and Th1 typical cytokine IFN‐γ expression in the induced CD4^+^ T cells. *n* = 6. Representative FCM plots (E) and statistical analysis (F) of Th2 cell transcription factor GATA‐3 and Th1 transcription factor T‐bet in the induced CD4^+^ T cells. *n* = 6. The data are presented as mean ± SEM. (*p < .05, **p < .01; ***p < .001, Student's test)

We have identified that CXCR4^+^ dNK cells preferentially express immunomodulatory molecules including IL‐4, IL‐10 and TGF‐β. Our analysis of cytokines produced by different decidual immune subgroups showed that more than 80% of IL‐4 production derives from NK cells (Figure S4). Further analysis showed that more than 50% of CXCR4^+^ dNK cells produce IL‐4, whereas less than 5% of CXCR4^–^ dNK cells produce IL‐4 (Figure 5A). Analysis of source of IL‐4 at maternal–foetal interface and in the peripheral blood showed that >80% of IL‐4 was produced by NK cells at maternal–foetal interface while ∼60% of IL‐4^+^ cells in the peripheral blood are CD3^+^CD56^–^ T cells (Figure 5B). These findings suggest that CXCR4^+^ dNK cells represent the main source of IL‐4 at maternal–foetal interface.

Given IL‐4 is essential for Th2 differentiation, we hypothesised that CXCR4^+^ dNK cells may influence the polarisation of CD4^+^ T cells. We therefore co‐cultured CD4^+^CD45RA^+^ naïve T cells isolated from peripheral blood with CXCR4^+^ dNK or CXCR4^–^ dNK cells, respectively. We found that the production of IL‐4 but not IFN‐γ was significantly upregulated when naïve T cells were co‐cultured with CXCR4^+^ dNK cells and this effect could be abrogated by the presence of IL‐4 neutralising antibodies (Figure 5C and D). In contrast, co‐culture with CXCR4^–^ dNK cells significantly upregulated production of IFN‐γ in CD4^+^ T cells but had little effect on IL‐4 production (Figure 5C and D). Furthermore, we found that co‐culture with CXCR4^+^ dNK cells significantly upregulated the Th2‐specific transcription factor GATA‐3 but had little effect on the Th1‐specific transcription factor T‐box transcription factor 21 (T‐bet) in CD4^+^ T cells, and these effects could be inhibited by IL‐4 neutralising antibodies (Figure 4E and F). In contrast, co‐culture with CXCR4^–^ dNK cells significantly upregulated T‐bet but had little effect on GATA‐3 in CD4^+^ T cells. We also examined the Treg‐specific transcription factor forkhead box P3 (Foxp3) in naïve T cells and found no obvious promotion of Treg differentiation by CXCR4^+^ or CXCR4^–^ dNK cells (Figure S5). Collectively, these results show that the IL‐4 production predominantly by CXCR4^+^ dNK cells endows these cells with the capacity to promote the differentiation of naïve T cells into a Th2 subtype.

### 3.5 CXCR4^+^ dNK cells with compromised Th2 inducing potential evokes abnormal CD4^+^T cells activation in patients with RM

**FIGURE 6:**
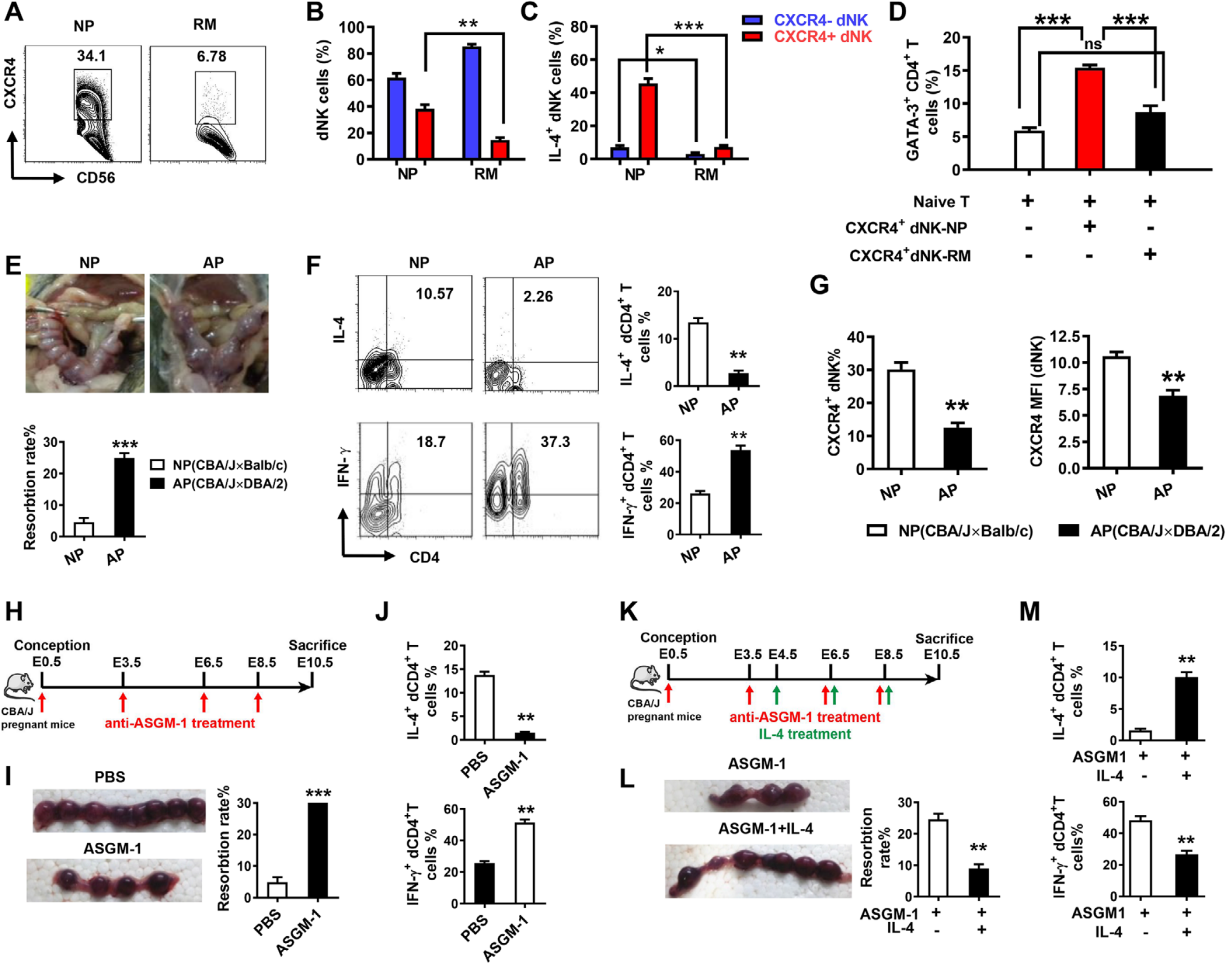
Dysfunctional CXCR4^+^ dNK cells are involved in Th1 response in miscarriages. (A and B) Percentage of CXCR4^+^ dNK cells in recurrent miscarriages (RM) and normal early pregnancy (NP). *n* = 6. (C) Percentage of IL‐4‐expressing CXCR4^+^ and CXCR4^–^ dNK cells in RM and NP (*n* = 6). (D) CXCR4^+^ dNK cells isolated from RM patients and NP were added to the Naïve T cell‐induced differentiation system. GATA‐3 was detected by FCM (*n* = 6). (E) Representative images of the foetus and embryo resorption rates of (normal pregnancy) NP and abortion‐prone (AP) mice (*n* = 6). (F) FCM analysis of IL‐4 and IFN‐γ produced by decidual CD4^+^ T cells in NP and AP mice (*n* = 6). (G) Percentage and mean fluorescence intensity (MFI) of CXCR4^+^ NK cells in NP and AP mice (*n* = 6). (H) Schema of NK‐deletion in normal pregnant mice. (I) Representative images of embryos and resorption rates of NP mice treated with PBS or anti‐ASGM‐1 (*n* = 6). (J) FCM analysis of IL‐4 and IFN‐γ generated by decidual CD4^+^ T in NP mice treated with PBS or anti‐ASGM‐1 (*n* = 6). (K) Schema of NK‐deletion in NP mice supplemented with rmIL‐4. (L) Representative images of embryos and the resorption rates in NK‐depleted pregnant mice administered PBS or rmIL‐4. (M) FCM analysis of IL‐4 and IFN‐γ produced by decidual CD4^+^ T in NK‐depleted pregnant mice administered with PBS or rmIL‐4 (*n* = 6). The data are shown as mean ± SEM. (*p < .05, **p < .01, ***p < .001; ns, no significance; Student's test)

We next assessed whether there were differences between CXCR4^+^ dNK cells derived from RM and NP women. Interestingly, there were significantly lower percentages of CXCR4^+^ dNK cells detected in RM women compared with NP women (Figure 6A and B). Moreover, both RM‐derived CXCR4^+^ and CXCR4^–^ dNK subsets expressed less IL‐4 than those from NP women, and this was particularly evident in the CXCR4^+^ dNK cell subset (Figure 6C). In addition, we found that CXCR4^+^ dNK cells from healthy NP potently promoted GATA‐3 expression in CD4^+^CD45RA^+^ naïve T cells after 5‐day induction (Figure 6D). However, this phenomenon was not observed in RM‐derived CXCR4^+^ dNK cells, suggesting that CXCR4^+^ dNK cells from RM patients become incapable to induce T‐cell GATA‐3 expression (Figure 6D).

A similar phenomenon was observed comparing normal pregnancy (BALB/c male mated with CBA/J female) with abortion‐prone (DBA/2 male mated with CBA/J female) mouse models (Figure 6E–G). FCM analysis of decidual cells showed that CD4^+^ T cells from the abortion‐prone mice produced a Th1 shift with much lowered IL‐4 but higher IFN‐γ than CD4^+^ T cells from normal pregnant mice (Figure 6F). This difference in CD4^+^T differentiation was limited to the decidua and not found in peripheral CD4^+^ T cells (Figure S6). Moreover, CXCR4 expression on dNK cells was decreased in abortion‐prone mice compared to normal pregnant mice (Figure 6G).

To confirm the direct regulation of dNK cells in CD4^+^ T‐cell differentiation in vivo, we deleted NK cells in normal pregnant CBA/J mice with anti‐asialo GM‐1 antibody (ASGM‐1) (Figure 6H–J). As expected, anti‐ASGM‐1 injected mice showed a sharp decrease in the percentage of dNK cells (from more than 30% to less than 5%) and pNK cells (from around 10% to less than 1%) compared to normal pregnant mice (Figure S7). We also observed that the embryo absorption rate of NK cell‐depleted mice was significantly higher than that of NP mice (Figure 6I). Decidual CD4^+^ T cells in NK cell‐depleted pregnant mice showed higher expression of Th1‐type cytokine IFN‐γ but lower expression of Th2‐type cytokine IL‐4 compared to decidual CD4^+^ T cells in normal pregnant mice (Figure 6J). However, these effects could not be observed in spleen CD4^+^ T cells (Figure S8). Furthermore, we supplemented exogenous cytokine IL‐4 into NK cell‐depleted pregnant mice to observe pregnancy outcomes and cytokine production from decidual CD4^+^ T cells (Figure 6K–M). Interestingly, along with decreased rates of embryo absorption, treatment with IL‐4 notably recovered Th2 bias in NK‐deleted pregnant mice at maternal–foetal interface (Figure 6L and M). These data suggest the NK cells contribute to Th2 bias in decidua via IL‐4; without NK cells, CD4^+^ T cells may differentiate towards a Th1 bias, potentially enhancing the inflammatory responses at the maternal–foetal interface and causing foetal loss.

### 3.6 CXCR4^+^ dNK cells exhibit therapeutic potential in relieving pregnancy loss via promoting Th2 shifts

**FIGURE 7:**
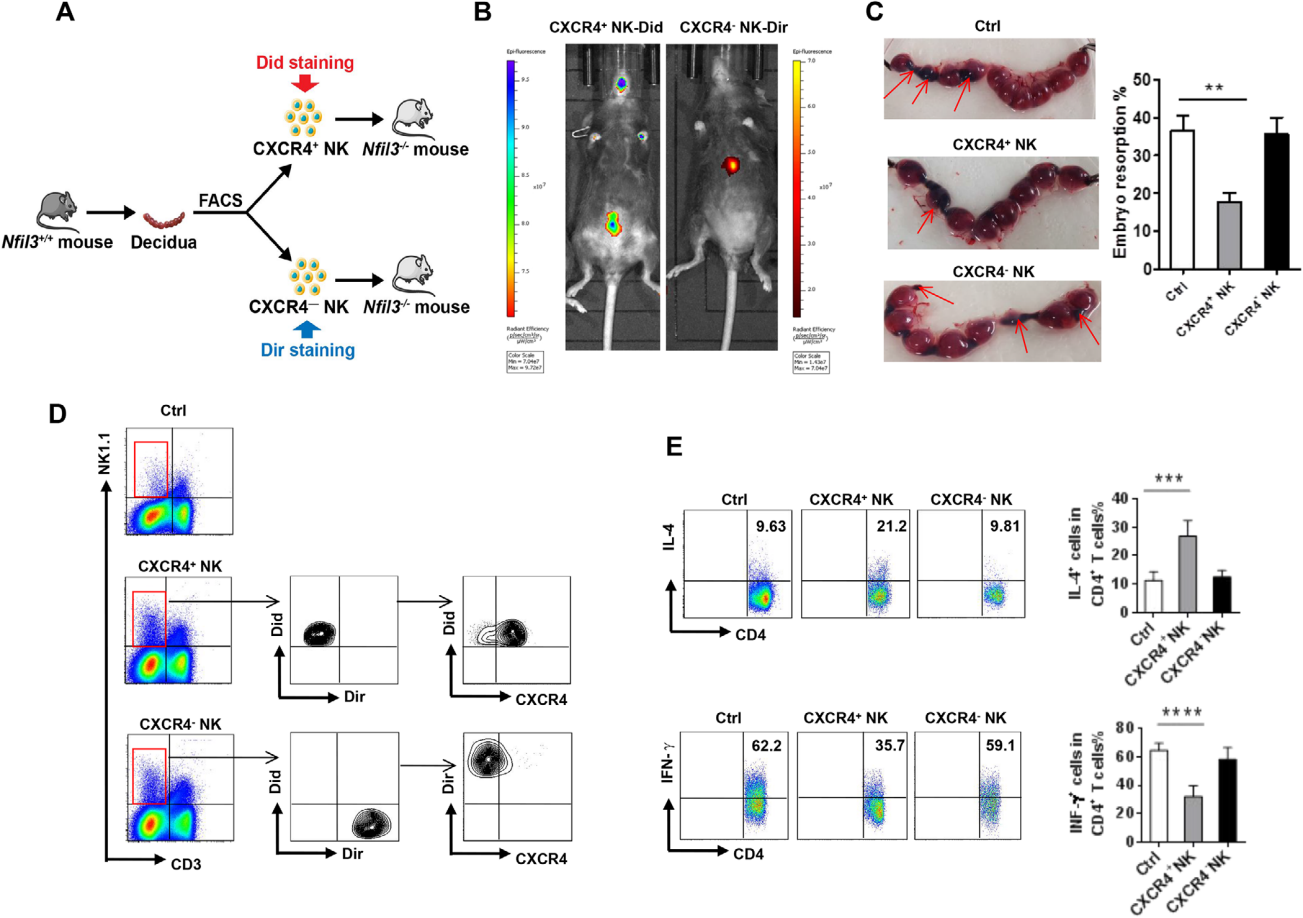
CXCR4^+^ NK cells alleviate pregnancy loss in NK‐deficient mice. (A) CXCR4^+^ and CXCR4^–^ NK cells sorted from C57BL/6 (Nfil3^+/+^) pregnant mice were fluorescently labelled and intravenously injected into Nfil3^−/−^ (NK‐deficient) pregnant mice at E7.5. (B) In vivo imaging of the transferred Did‐CXCR4^+^ NK and Dir‐CXCR4^–^ NK in recipient mice. (C) Representative embryos pictures in uterus of Nfil3^−/−^ pregnant mice receiving the indicated NK cell populations. (D) FCM analysis of the Did‐labelled CXCR4^+^ NK and Dir‐labelled CXCR4^–^ NK within the decidual immune cells of Nfil3^−/−^ pregnant mice receiving the indicated NK cell populations. (E) FCM showing the percentages of IL‐4^+^CD4^+^T cells and IFN‐γ^+^CD4^+^T cells in control Nfil3^−/−^ mice or those receiving the indicated NK cell populations. The data are shown as mean ± SEM. (**p < .01, ***p < .001, ****p < .0001; Student's test)

To further explore whether the immunomodulatory CXCR4^+^ dNK subset could be used to overcome pregnancy loss, NK cell genetically deleted mice, Nfil3^–/–^ mice were used. We isolated CXCR4^+^ dNK cells and CXCR4^–^ dNK cells from Nfil3^+/+^ pregnant mice and differentially stained these subsets with DilC18(5) (Did) or DilC18(7) (Dir), respectively, before adoptively transfer into pregnant Nfil3^–/–^ mice at E7.5 (Figure 7A). In vivo imaging after 3 days showed Did‐stained CXCR4^+^ dNK cells were predominantly enriched in uterine region while few Dir‐stained CXCR4^–^ dNK cells were found in the uterus or surrounding areas (Figure 7B). Moreover, adoptive transfer of CXCR4^+^ dNK, but not CXCR4^−^ dNK, substantially decreased the embryo resorption rate in recipient Nfil3^–/–^ mice (Figure 7C). Further FCM analysis of the decidua of Nfil3^–/–^ mice confirmed that transferred dNK cells successfully homed to the pregnant uterus with lesser numbers of the CXCR4^–^ dNK cells relative to CXCR4^+^ NK cells (Figure 7D). Moreover, we also found after administration of Nfil3^–/–^ mice with CXCR4^+^ dNK cells, CD4^+^ T cells displayed significantly higher expression of IL‐4 but lower expression of IFN‐γ than those administered CXCR4^–^ dNK cells (Figure 7E). Together, these data indicate promising potential for CXCR4^+^ dNK cells as cell‐based immunotherapy for pregnancy failure.

## 4 DISCUSSION

The most prominent feature of early pregnancy is that large quantity of NK cells accumulated at maternal–foetal interface, which suggests that NK cells play an important part in the maintenance of pregnancy. The origin and exact function of the accumulated NK cells, however, remains incompletely understood. In the current study, RNA sequencing analysis of peripheral and decidual NK cells during early pregnancy identified an immunomodulatory dNK subset characterised as CXCR4^+^CD56^bright^dNK. Investigations the origins of this subset established that CXCR4^+^CD56^bright^dNK cells can be recruited from peripheral blood and be reprogramed by trophoblasts, thus actively participating in the establishment of immune‐tolerance during early pregnancy. Proof of adoptive transfer experiments showed CXCR4^+^ dNK cells have great therapeutic potential in treatment of pregnancy failures such as miscarriages.

Through the menstrual cycle, the number of uterine NK (uNK) cells is constantly changing. A dramatic increase of uNK cells can be observed around the time of implantation, that is days 6 and 7 after the luteinising hormone (LH) surge in humans. The large population of uNK cells will continue to persist if successful pregnancy is established and these cells are renamed decidual NK after decasualisation. At the early stage of pregnancy, uNK even account for 70% of the total immune cells at maternal–foetal interface; however, uNK cell numbers gradually decline as gestation progresses. While the function of uNK cells in recurrent miscarriage is suspected to be crucial, previous investigations have not provided definitive answers. On one hand, independent studies have shown increases in the numbers of CD56^+^ uNK cells in women with RM,^36^, ^37^ while others were unable to show any differences.^38^ On the other hand, research focused on the predictive value of peri‐implantation uNK cell number in RM women failed to establish significant differences in their pregnancy outcomes.^39^ Nonetheless, the unique abundance and time‐related quantitative changes in NK cells in the early stages of pregnancy remains compelling. We therefore speculated that the true significance of NK cells may lie in specific cell subpopulations and that alterations in NK cell subsets may be responsible for pregnancy loss.

The expression of CXCR4 on NK cells during early pregnancy was explored in this research where we characterised the phenotype and function of a novel decidual NK subset, CXCR4^+^CD56^bright^ dNK cells (abbreviated to CXCR4^+^ dNK). Distinguished from CXCR4^–^ dNK, CXCR4^+^ dNK represent a unique NK subset with low activity and cytotoxicity but high anti‐inflammatory capacity in decidua. Interestingly, single‐cell reconstruction in human early pregnancy suggested a subset of dNK3 which characterised by the expression of CD160, CD161 and CD103, but not CD127. The dNK3 subset was proposed to regulate extravillous trophoblast (EVT) invasion and their scRNA‐seq data showed that dNK3 expressed low levels of KIRs (KIR2DL1, KIR2DL2 and KIR2DL3) and cytotoxic genes such as PRF1 (perforin 1), GNLY (granulysin), GZMA (granzyme A) and GZMB (granzyme B) and lastly, high levels of CXCR4.^15^ This strongly supports our findings and suggests that it is reasonable to identify CXCR4^+^ dNK cells as a distinct dNK subset which is essential for establishing an immune‐tolerant environment that permits embryo survival without immune‐attack.

Our previous studies together with other reports have shown that human first‐trimester trophoblasts could recruit CD56^bright^CD16^–^ NK to decidua by means of expressing and secreting CXCL12.^31^, ^40^ It has also been showed that the CXCR4/CXCL12 signal is essential for NK development.^41^ Exploring the origin of the CXCR4^+^CD56^bright^ dNK subset we found that CXCR4^+^ pNK cells can be recruited by trophoblasts via CXCL12. Moreover, we found CXCR4^+^ dNK cells express higher levels of anti‐inflammatory cytokines (such as IL‐4 and IL‐10), lower levels of pro‐inflammatory cytokines (such as INF‐γ and TNF‐α) as well as perforin. It is also suggested by our results that CXCR4‐mediated immune‐regulation by dNK during pregnancy is enhanced by CXCL12. Previous studies have reported the effects of CXCR4/CXCL12 on NK cells regarding their chemotaxis, distribution and development.^42^, ^43^, ^44^ However, while CXCR4/CXCL12 regulates the functions of vascular endothelial cells and tumour cells,^26^, ^45^, ^46^ their influence on NK cell function is still under debate. Our results indicate that CXCR4/CXCL12 not only affects the distribution of NK cells during early pregnancy, but also regulates NK function at maternal–foetal interface. Thus, the two distinct NK subgroups defined by the expression of CXCR4 are helpful in depicting the plasticity of decidual NK cells.

We noticed that CXCR4 expression was negatively related to CD56 expression when we compared their expression on pNK cells and dNK cells as well as the CXCR4^+^ dNK and CXCR4^–^ dNK cells. CD56^dim^NK cells are considered more mature than CD56^high^ NK cells, thereby proposing that CXCR4 represents a late‐ stage differentiation marker of NK cells. How the mutually restricted expression of CD56 and CXCR4 occurs is unclear but is likely governed by endogenous regulatory factors. Our data indicated that trophoblasts act as exogenous positive regulators of CD56 and CXCR4 to recruit and reprogram pNK cells to dNK cells, leading to upregulation of CD56 and downregulation of CXCR4. Thus, the transformation of peripheral NK to decidua NK phenotype might be affected by the combined effect of exogenous regulatory factors such as trophoblasts at maternal–foetal interface and the endogenous regulatory network of NK cells.

Traditionally classified as innate immune cells, NK cells could rapidly react against target cells in the absence of prior sensitisation.^47^ Currently, increasing evidence suggests that they also exert other regulatory functions. For instance, IL‐10‐secreting NK1 and TGF‐β‐secreting NKr cells may play important roles in immune modulation especially the tolerance of transplants and pregnancy.^48^, ^49^ Moreover, a subset of NK17/NK1 cells characterised by the expression of CCR4 and CD56 along with the IFN‐γ and IL‐17 production was identified from IL‐2 activated normal human peripheral blood,^50^ and NK22 cells were found to have important regulatory roles in mucosal immunity.^51^ CD56^bright^CD27^+^ NK cells were found to promote immune tolerance by suppressing inflammatory Th17 cells via IFN‐γ secretion in successful pregnancy.^52^ In our previous work, we found that CD56^bright^CD25^+^ dNK are the main source of TGF‐β and are essential for immune tolerance during early human pregnancy.^18^ Together, these findings show that NK cells are an important source of cytokines, which are extensively involved in intercellular communication. Here, we provide evidence that CXCR4^+^ dNK subset is the main source of IL‐4 at maternal–foetal interface and plays a key role in predominant Th2 differentiation in early pregnancy. In fact, the immune microenvironment of the decidua prevents inflammatory responses. CXCR4^+^ dNK cells‐promote Th2 bias conducive to restraining maternal immune responses in favour of tolerating the allogenic foetus. This immunoregulatory role of CXCR4^+^ dNK cells was further confirmed in mouse models of normal pregnancy and spontaneous abortion together with studies in NK cell‐depleted mouse models. Clinically, we found the percentage of CXCR4^+^ dNK cells decreased in RM patients and their expression of IL‐4 as well as IL‐4‐mediated Th2 differentiation was dampened. Therefore, the CXCR4^+^ dNK subset possessing immunomodulatory functions plays a key role in normal pregnancy and deficient CXCR4^+^ dNK with compromised Th2 induction potential are likely to be primary mediators of RM.

The possibility of targeting the CXCR4/CXCL12 signalling axis is currently well progressed with different therapeutic approaches already available for clinical applications. The small molecule CXCR4 antagonist, AMD3100 (plerixafor), is the most frequently used drug in clinical trials for gastrointestinal solid tumours targeting the CXCR4/CXCL12 axis.^53^ A novel CXCR4 agonist, SDV1a, was designed and could effectively enhance the therapeutic effect of transplanted stem cells.^54^ Moreover, chimeric antigen receptor (CAR) NK cells overexpressing CXCR4 were shown effective against tumours.^55^ Here, we clarified the role of CXCR4/CXCL12‐mediated immunoregulation of decidual NK cells in early pregnancy. More importantly, we established the effectiveness of adoptive transfer of CXCR4^+^ dNK cells in the treatment mouse models of pregnancy loss. In Nfil3^–/–^ mice, NK cells are absent in periphery and are severely reduced in lungs, spleen, liver and uterus.^34^, ^56^, ^57^ Pregnancy loss of Nfil3^–/–^ mice was obviously relieved as well as the pro‐inflammatory Th1 response was inhibited after received CXCR4^+^ dNK cells. Thus, our study opens the possibility of two distinct treatment approaches for RM. First, CXCR4 agonists could be used as immunomodulatory agents to alter the recruitment and function of CXCR4^+^ dNK cells to provide a more permissive state to establish successful pregnancies. Second, the adoptive transfer of CXCR4^+^ dNK cells as a cell‐based therapy in RM patients could also be considered. However, the disease of RM especially idiopathic disease is heterogeneous and altered maternal–foetal immunotolerance may represent only one of the causes. It should also be mentioned that the RM samples were collected in this study occurred after foetal demise, and the inflammatory responses caused by foetal death itself cannot be excluded.

**FIGURE 8:**
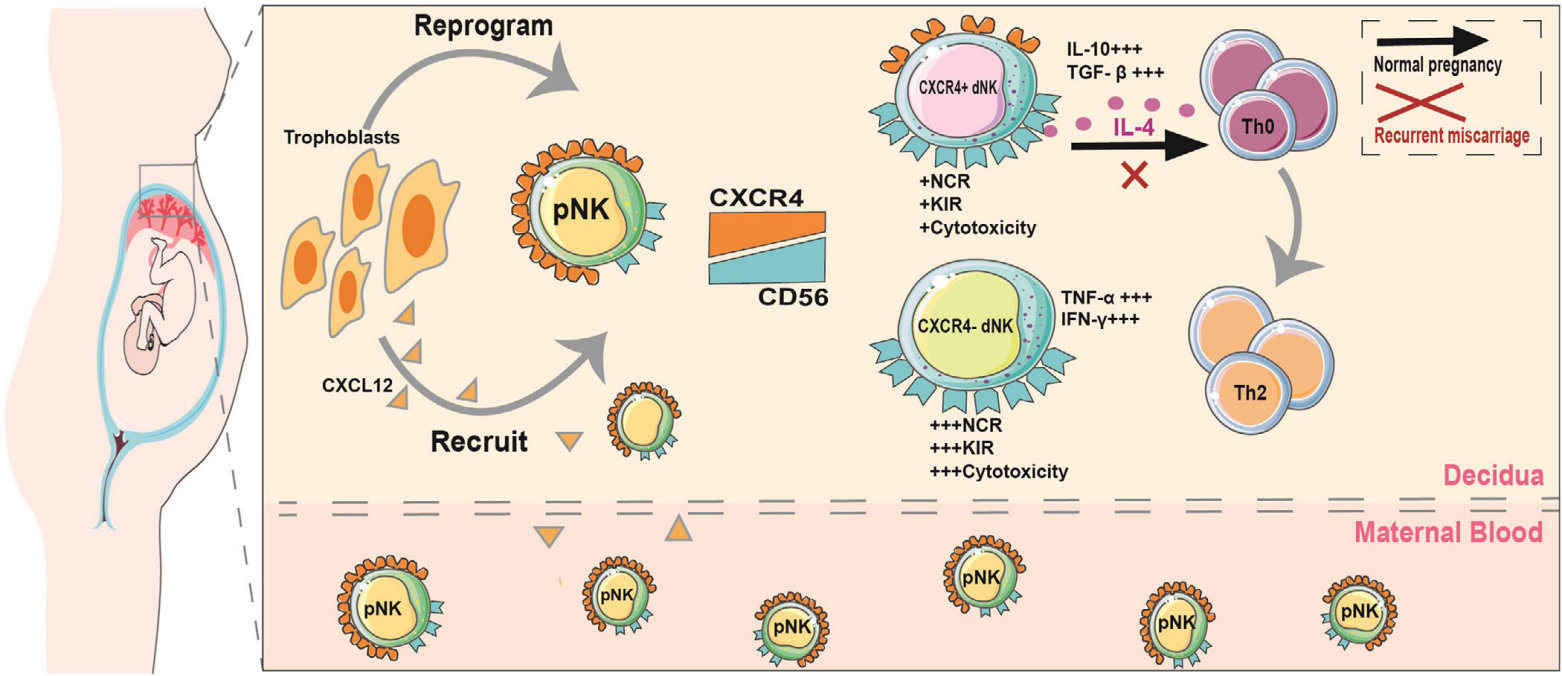
Schematic diagram illustrating the origin, phenotype and function of CXCR4^+^CD56^bright^ dNKs in maternal–foetal immune tolerance during early pregnancy. Trophoblasts attract peripheral CXCR4^+^ NK through secreting CXCL12. Trophoblasts further upregulate CD56 and downregulate CXCR4 expression of CXCR4^+^ pNK to adopt a dNK‐similar phenotype. CXCR4^+^CD56^bright^ dNK are the main source of IL‐4 at maternal–foetal interface, which induce CD4^+^CD45RA^+^ naïve T cells to preferentially differentiate to Th2‐type cells, involving in the maintenance of normal pregnancy. In miscarriage, the number and IL‐4‐prodution of CXCR4^+^CD56^bright^ NK cells in the decidua are compromised, which fail to promote Th2 bias at maternal–foetal interface

In summary, our work identified a subset of CXCR4^+^CD56^bright^ dNK cells that represents low activity, reduced cytotoxicity but high capacity of anti‐inflammation. We depicted its origin and phenotypic transformation under the instruction of trophoblasts. In addition, we defined the immunoregulatory capacity of CXCR4^+^ dNK subset to induce Th2 differentiation via IL‐4 to prevent inflammatory responses within decidual environment (Figure 8). Targeting the CXCR4/CXCL12 signalling of dNK cells might bring us new perspective and therapeutic options for pregnancy failures such as RM.

## CONFLICT OF INTEREST

The authors declare that they have no competing interests.

## Supporting information



Supporting Information
