# Supplementary material for: Decidual CXCR4+CD56brightNK cells as a novel NK subset in maternal–foetal immune tolerance to alleviate early pregnancy failure
Source: Clin Transl Med. 2021 Oct 14;11(10):e540. doi: 10.1002/ctm2.540 (PMC8516340; doi:10.1002/ctm2.540)
Supplement: Supplementary file 1 — Supporting Information [file CTM2-11-e540-s001.doc]

Supplementary Information

**Decidual CXCR4^+^CD56^bright^NK cells as a novel NK subset in maternal-fetal immune tolerance to alleviate early pregnancy failure**

**Tao et al.**

**
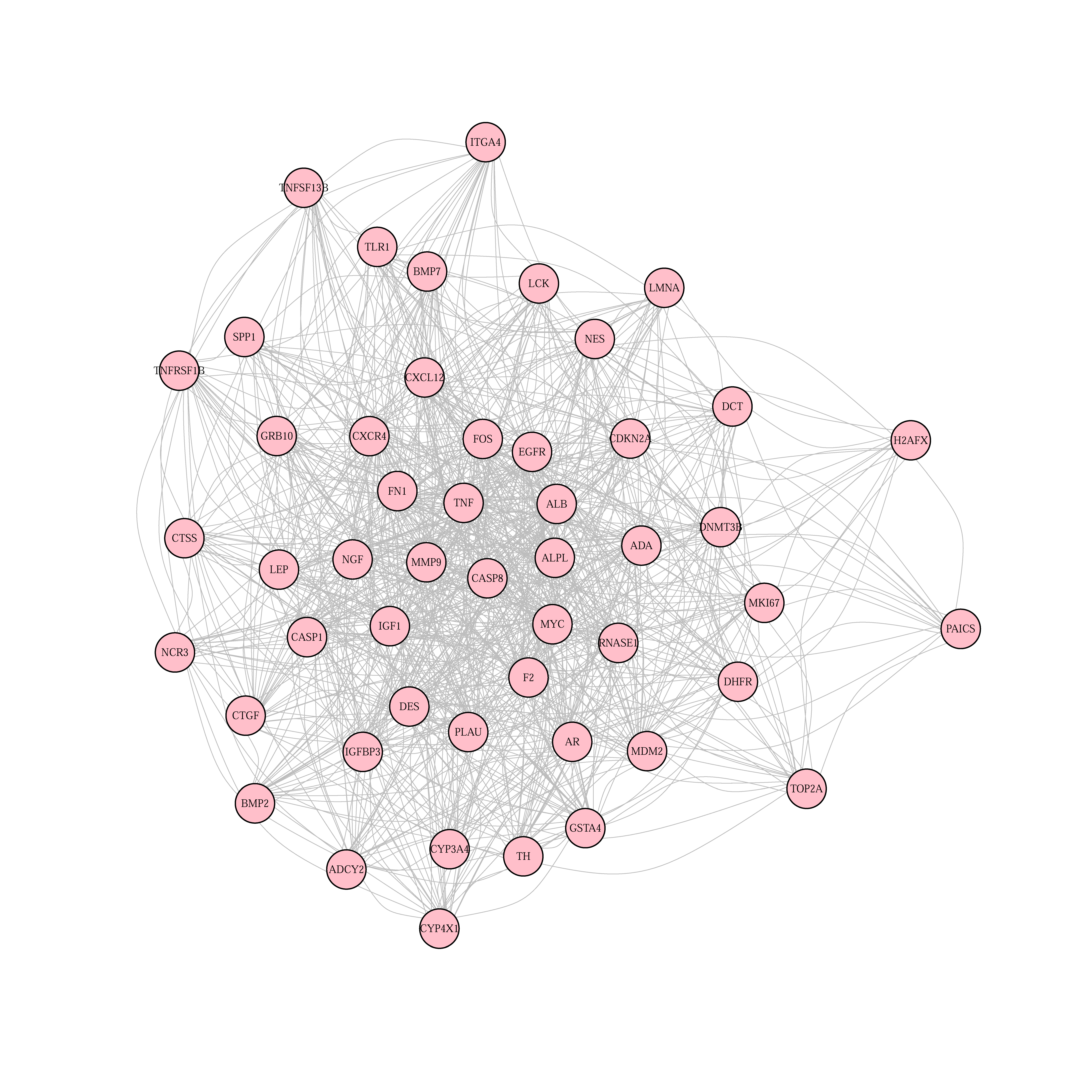
**

**Supplementary Figure 1. The PPI network of DEGs identified between dNK and pNK cells.** Based on the STRING database and Cytoscape software, relevant PPI of DEGs identified between dNK and pNK cells were constructed and visualized that contained top 50 networks. PPI, protein-protein interaction network; DEG, differentially expressed gene.


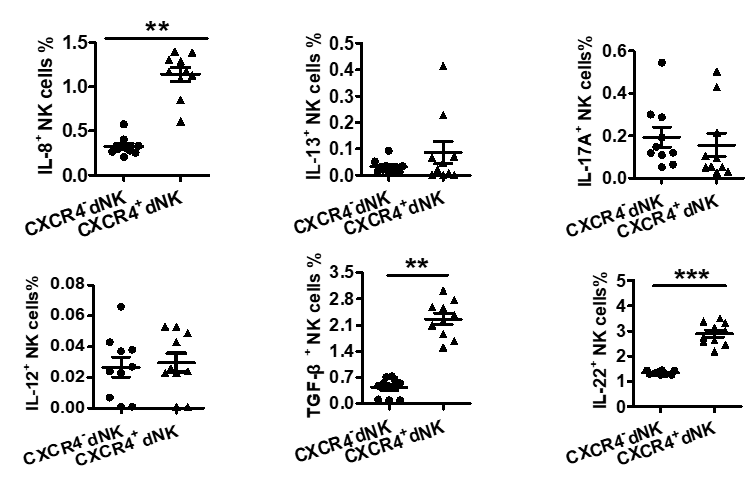


**Supplementary Figure 2. Cytokine expression in CXCR4^+^ and CXCR4^-^dNK cells.** FCM analyze the percentage of various cytokines expression in CXCR4^+^ and CXCR4^-^dNK cells. n=10 Data are presented as the means ± SEM. ** P<0.01.


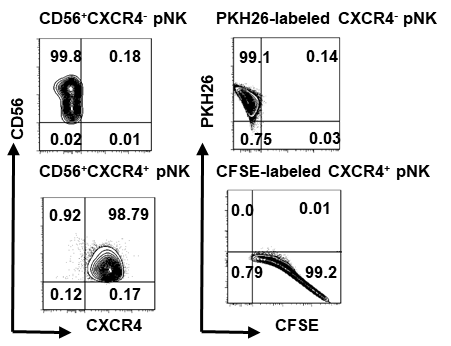


Supplementary Figure 3. Identification of CXCR4^+^ and CXCR4^-^pNK cells before and after CSFE and PKH26 labeling. Representative density plots of MACS-sorted CXCR4^+^ and CXCR4^-^ pNK cells (left column). Representative density plots of CSFE-labeled CXCR4^+^pNK cells and PKH26-labeled CXCR4^-^pNK cells (right column).


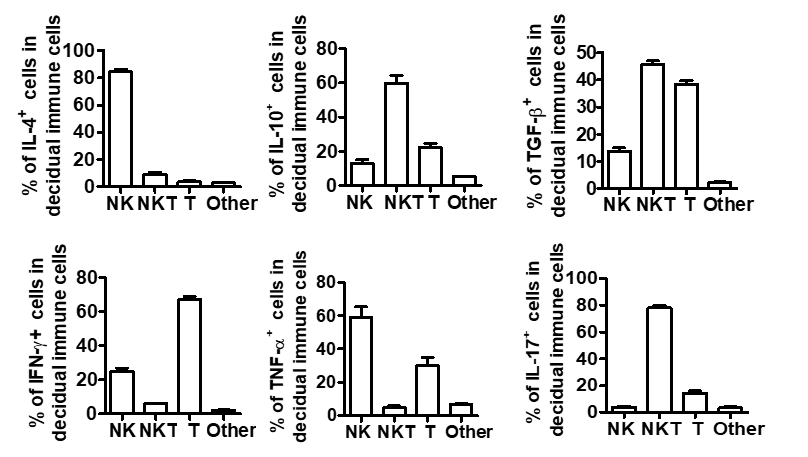


**Supplementary Figure 4.** **Cytokines production by different immune cells at the maternal-fetal interface.** FCM analysis shows different sources of various cytokines from decidual immune cells at the maternal-fetal interface.

**Supplementary Figure 5.** **CXCR4^+^dNK cells had no direct effect on Treg specific transcription factors Foxp3.** Percentage analyses showed that CD56^bright^CXCR4^+^NK cells t had little effect on Treg specific transcription factors Foxp3 of CD4^+^CD45RA^+^ naïve T cells. n=6. Data are presented as the means ± SEM.


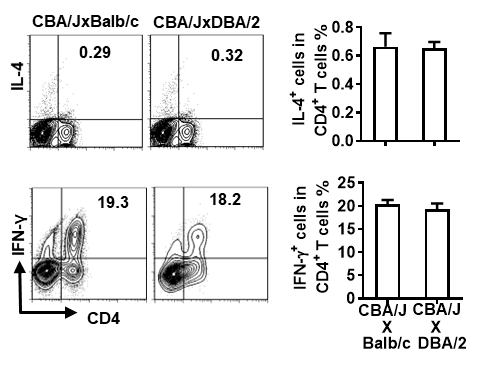


Supplementary Figure 6. Comparation of Th1/Th2 cytokine production by spleen CD4^+^T cells in normal pregnant and abortion-prone mice. There was no difference of IL-4 and IFN-γ production in spleen CD4^+^T cells from normal pregnant and abortion-prone mice. n=6. Data are presented as the means ± SEM.


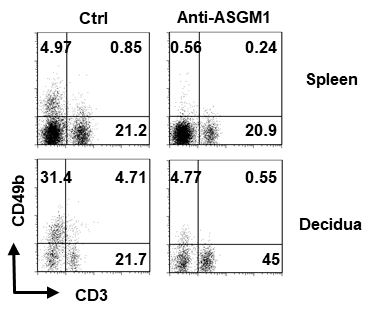


**Supplementary Figure 7. The efficacy of NK cell depletion by anti-ASGM-1.** Female CBA/J mice were mated in natural cycling with male BALB/c mice. Pregnant CBA/J mice were injected with anti-ASGM-1 at E0.5, E3.5, E6.5, and E8.5 and euthanized on E10.5. CD3 and CD49b expression was assessed using FCM analysis, which showed the efficacy and specificity of NK cell depletion by anti-ASGM-1**.**


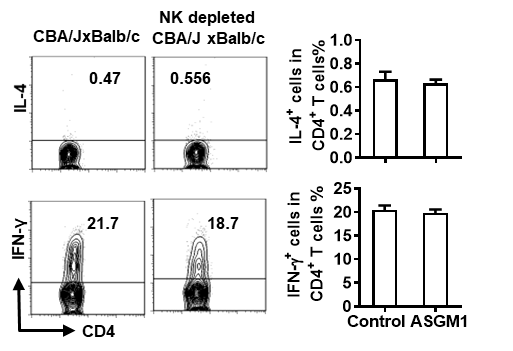


**Supplementary Figure 8.** **Deletion of NK cell in pregnant CBA/J mice exerts no effect on IL-4 and IFN-γ production in spleen CD4^+^T cells from pregnant mice.** n=6. Data are presented as the means ± SEM.

**Supplementary Table S1 Demographics and clinical characteristics of the patients with recurrent miscarriage**

|  | RM (n=45) |
| --- | --- |
| Age(years) | 29.1 ± 6.80 |
| Gestational age(weeks) | 7.45 ± 1.44 |
| Gravity | 3.93 ± 0.18 |
| Parity | 0.13 ± 0.05 |
| Miscarriages | 2.78 ± 0.13 |
| 2 | 21 (46.7%) |
| 3 | 15 (33.3%) |
| 4 | 7 (15.6%) |
| ≥5 | 2 (4.4%) |

Data are mean ± SEM.
RM: recurrent miscarriage

**Supplementary Table S2 Demographics and clinical characteristics of the women with normal pregnancy (decidua and peripheral blood tissue)**

|  | NP (n=205) |
| --- | --- |
| Age(years) | 29.15 ± 5.27 |
| Gestational age(weeks) | 8.35 ± 1.12 |
| Gravity | 2.31 ± 0.05 |
| Parity | 1.14 ± 0.02 |

Data are mean ± SEM.
NP: normal pregnancy

**Supplementary Table S3 Demographics and clinical characteristics of the women with normal pregnancy (villous tissues)**

|  | NP (n=165) |
| --- | --- |
| Age(years) | 27.50 ± 3.42 |
| Gestational age(weeks) | 8.28 ± 1.25 |
| Gravity | 2.39 ± 0.06 |
| Parity | 1.12 ± 0.02 |

Data are mean ± SEM.

NP: normal pregnancy

**Supplementary Table S4 Antibodies used**

| **Antibody** | **Supplier** | **Catalogue number** |
| --- | --- | --- |
| IL-4 neutralizing antibody | eBioscience | 16-7048-81 |
| CD3 | eBioscience | 14-0032-82 |
| CD28 | eBioscience | 14-0281-82 |
| CXCR4 | eBioscience | 14-9991-82 |
| FITC, PE/Cy7 anti-human CD3 | Biolegend | 317306, 100331N1 |
| APC, PE/Cy7 anti-human CD56 | Biolegend | 362504, 105601N1 |
| APC anti-human CD16 | Biolegend | 302012 |
| PE/Cy7 anti-human CD4 | Biolegend | 100421N1 |
| PE anti-human CD45RA | Biolegend | 304108 |
| PE, APC anti-human CD25 | Biolegend | 302606, 302610 |
| FITC anti-human CD94 | Biolegend | 305504 |
| PE, APC anti-human CXCR4 | Biolegend | 306506, 306510 |
| APC anti-human NKp30 | Biolegend | 325210 |
| APC anti-human NKp44 | Biolegend | 325110 |
| APC anti-human NKp46 | Biolegend | 331918 |
| PE anti-human KIR2DL1 | Biolegend | 374904 |
| PE anti-human KIR3DL1 | Biolegend | 312708 |
| APC anti-human PD-1 | Biolegend | 621610 |
| APC anti-human CTLA-4 | Biolegend | 349908 |
| APC anti-human IL-4 | Biolegend | 500812 |
| PE anti-human IL-8 | Biolegend | 511408 |
| APC anti-human IL-10 | Biolegend | 501410 |
| FITC anti-human IL-12 | Biolegend | 501804 |
| APC anti-human IL-13 | Biolegend | 501907 |
| PE anti-human IL-17A | Biolegend | 512306 |
| APC anti-human IL-22 | Biolegend | 366706 |
| APC anti-human CD27 | Biolegend | 356409 |
| APC anti-human CD69 | Biolegend | 310909 |
| APC anti-human Perforin | Biolegend | 308112 |
| APC anti-human GraB | Biolegend | 396408 |
| APC anti-human INF-γ | Biolegend | 502512 |
| APC anti-human TNF-α | Biolegend | 502912 |
| PE anti-human TGF-β | Biolegend | 399704 |
| APC anti-human T-bet | eBioscience | 17-5825-82 |
| APC anti-human GATA-3 | eBioscience | IC63301A025 |
| PE anti-human FOXP3 | Biolegend | 320107 |
| FITC anti-mouse CD3 | Biolegend | 100204 |
| FITC anti-mouse CD4 | Biolegend | 130308 |
| FITC, PE/Cy7 anti-mouse CD49b | biolegend | 103504, 103518 |
| APC anti-mouse CXCR4 | Biolegend | 146508 |
| PE anti-mouse IL-4 | Biolegend | 504104 |
| PE anti-mouse INF-γ | Biolegend | 505808 |
| PE anti-mouse T-bet | eBioscience | 12-5825-82 |
| PE anti-mouse GATA-3 | eBioscience | 12-9966-42 |

**Supplementary Table S5 Abbreviations**

Anti-ASGM-1-anti-asialo GM-1

CAR-chimeric antigen receptor

CD-Cluster of differentiation

CFSE- Carboxyfluorescein diacetate succinimidyl ester

CXC3CR1-CX3C motif chemokine receptor 1

CXCL12-chemokine C-X-C motif ligand 12

CXCR4-CXC chemokine receptor 4

DETs-differential gene transcripts

DICs-decidual immune cells

DMEM-Dulbecco’s Modified Eagle Medium

dNK-decidual NK

EVT-extravillous trophoblast

FACS-Fluorescence activated Cell Sorting

FBS- fetal bovine serum

FCM-Flow Cytometry

FITC- Fluorescein Isothiocyanate

Foxp3-Forkhead box P 3

FPKM- Fragments Per Kilobase per Million

GA-gestational age

GATA3 -GATA binding protein 3

GNLY -granulysin

GZMA -granzyme A

GZMB -granzyme B

HBSS-Hank’s Balanced Salt Solution

HEPES- 4-(2-Hydroxyethyl)piperazine-1-ethanesulfonic acid

IFN-interferon

IL- interleukin

IL1B-interleukin 1 beta

IVIg-intravenous immunoglobulin

KLRB1 – killer cell lectin like receptor B1

LIT-lymphocyte active immunotherapy

MACS-magnetic activated cell sorting

NCAM1 – neural cell adhesion molecule 1

NFIL3 – nuclear factor, interleukin 3 regulated

NK-Natural killer

NKT-natural killer T

NP-normal pregnancy

PBMC-peripheral blood mononuclear cell

PBS-phosphate buffer saline

PE-phycoerythrin

PMA-phorbol myristate acetate

pNK-peripheral NK

PRF1 -perforin 1

RM-Recurrent miscarriage

RPMI- Roswell Park Memorial Institute

TCM-conditioned medium

TGF -Transforming growth factor

TNF - Tumor necrosis fact
